# Modeling the Age-Associated Decrease in Mortality Rate for Congenital Anomalies of the Central Nervous System Using WHO Metadata From Nine European Countries

**DOI:** 10.3389/fneur.2018.00585

**Published:** 2018-07-19

**Authors:** Josef Dolejs, Helena Homolkova, Petra Maresova

**Affiliations:** ^1^Department of Informatics and Quantitative Methods, University of Hradec Králové, Hradec Králové, Czechia; ^2^Department of Pediatric Neurosurgery, Charles University, Thomayer's Teaching Hospital, Prague, Czechia; ^3^Department of Economics, University of Hradec Králové, Hradec Králové, Czechia

**Keywords:** mortality rate, age, congenital anomalies of the central nervous system, WHO database, childhood

## Abstract

**Background:** In humans, the mortality rate dramatically decreases with age after birth, and the causes of death change significantly during childhood. In the present study, we attempted to explain age-associated decreases in mortality for congenital anomalies of the central nervous system (CACNS), as well as decreases in total mortality with age. We further investigated the age trajectory of mortality in the biologically related category “diseases of the nervous system” (DNS).

**Methods:** The numbers of deaths were extracted from the mortality database of the World Health Organization (WHO) for the following nine countries: Denmark, Finland, Norway, Sweden, Austria, the Czech Republic, Hungary, Poland, and Slovakia. Because zero cases could be ascertained over the age of 30 years in a specific age category, the Halley method was used to calculate the mortality rates in all possible calendar years and in all countries combined.

**Results:** Total mortality from the first day of life up to the age of 10 years and mortality due to CACNS within the age interval of [0, 90) years can be represented by an inverse proportion with a single parameter. High coefficients of determination were observed for both total mortality (*R*^2^ = 0.996) and CACNS mortality (*R*^2^ = 0.990). Our findings indicated that mortality rates for DNS slowly decrease with age during the first 2 years of life, following which they decrease in accordance with an inverse proportion up to the age of 10 years. The theory of congenital individual risk (TCIR) may explain these observations based on the extinction of individuals with more severe impairments, as well as the bent curve of DNS, which exhibited an adjusted coefficient of determination of ${\bar{R}}^{2}$ = 0.966.

**Conclusion:** The coincidence between the age trajectories of all-cause and CACNS-related mortality may indicate that the overall decrease in mortality after birth is due to the extinction of individuals with more severe impairments. More deaths unrelated to congenital anomalies may be caused by the manifestation of latent congenital impairments during childhood.

## Introduction

Age is among the most important determinants of health outcomes. Mortality rate is an irreplaceable indicator of community health, and it is well known that mortality increases exponentially with age after age 35 [e.g., (1–10)]. This increase in mortality rate with age can be attributed to biological processes [e.g., (11–15)], and is typically interpreted as a manifestation of aging [e.g., (16–19)]. Statistically, age can be considered a deterministic variable in this exponential relationship because coefficients of determination may be higher than 0.99 (4, 6, 20).

Significantly faster changes in mortality rate with age occur during childhood. Mortality rates dramatically decrease with age in developed countries up to the age of 10 years (5, 8–10, 21). This decrease is associated with age-based changes in causes of death during childhood (22–25). Individuals with more severe impairment typically die during the first hours or days of life, and the majority of these severe cases are classified as congenital anomalies or other impairments originating in the perinatal period. However, the proportion of severe congenital anomalies and impairments originating in the perinatal period is very small and decreases to zero as age increases (24). Investigating the age trajectories of mortality may help to elucidate whether the sequential exclusion of individuals with more severe impairments is responsible for the steep decrease in mortality rates with age. In addition, analyzing the shape of these trajectories via parametric modeling may improve our understanding of the biological processes responsible for the steep decrease in mortality during childhood.

Data extracted from the World Health Organization (WHO) mortality database (26) have revealed that age-based decreases in mortality rates after birth are significantly faster than subsequent increases in adulthood. The WHO database uses the following age intervals: [0, 24) h, [1, 7) days, [7, 28) days, [28, 365) days, and 5-year age intervals after the age of 5 years. For example, in Denmark, the mortality rate for both sexes 0–24 h after birth was 56,072 per 100,000 living persons per year in 1994. The specific age trajectory of mortality reaches the minimal value (13.0 per 100,000 living persons per year) during the age interval of [10, 15) years. Mortality rates increase after the minimum, reaching 23,003 deaths per 100,000 living persons per year in the last age interval of the database (i.e., [90, 95) years). Figures in other European countries and in other calendar years are similar. These three outer mortality rates and their ratios are presented for nine countries of the European Union (EU) in Table 1. Data are presented for the two calendar years representing the first and last years for which data are available for a specific country. In different countries, the International Classification of Diseases (ICD10) classifications (27) and four important age categories ([0, 24) h, [1, 7) days, [7, 28) days, [28, 365) days) are used in different calendar period (Table 1).

**Table 1:** Total mortality rate per 100,000 living persons per year in the three extreme age categories.

**Country**	**Year**	**The 1st day**	**The minimal value**	**M (years)**	**90–95 years**	**Size of population**	**Ratio A**	**Ratio B**
Denmark	1994	56,071	13.0	15	23,003	5,201,016	4,320	1,772
Denmark	2009	28,786	6.6	10	20,009	5,491,215	4,406	3,062
Finland	1996	36,675	13.7	5	24,885	5,117,510	2,695	1,828
Finland	2011	16,811	5.3	10	19,169	5,466,882	3,215	3,665
Norway	1996	56,128	12.0	10	22,835	4,377,175	4,712	1,917
Norway	2011	25,520	3.5	10	19,673	5,197,587	7,417	5,718
Sweden	1997	44,921	11.1	15	22,732	8,889,449	4,058	2,053
Sweden	2010	27,171	6.1	4	21,421	9,779,521	4,461	3,517
Austria	2002	47,444	10.2	10	22,083	8,168,634	4,685	2,180
Austria	2011	29,399	5.0	5	17,338	8,689,683	5,953	3,511
Czech	1994	110,440	24.2	15	40,731	10,328,577	4,571	1,724
Rep.	2015	37,853	7.4	4	21,979	10,636,473	5,151	2,990
Hungary	1996	128,436	19.7	5	27,794	10,255,681	6,542	1,415
Hungary	2010	73,429	8.3	10	18,176	9,885,318	8,933	2,211
Poland	1999	90,700	19.6	10	23,357	38,648,841	4,646	1,196
Poland	2011	39,635	9.8	10	17,870	38,519,432	4,064	1,832
Slovakia	1996	121,250	22.5	15	24,041	5,371,977	5,400	1,070
Slovakia	2009	89,761	10.0	4	18,151	5,439,590	9,012	1,822

*The 10th ICD revision (27) and the four important age categories [0, 24) h, [1, 7) days, [7, 28) days, [28, 365) days are used in different countries in different calendar periods. The outer years of the periods are in Table 1. The first day represents the first possible age interval and [90, 95) years is the last age interval in the WHO database (26). Total mortality rate reaches its minimal value usually in the age interval 5–10 years. **M** is upper age of the age category where mortality rate reaches the minimal value. Ratio A is the ratio of the mortality rate in the 1st day to the minimal value of mortality rate, Ratio B is the ratio of the mortality rate in the age interval 90–95 years to the minimal value of mortality rate*.

The total mortality rate decreases three orders of magnitude with age in the short age interval of [0, 10) years (see “Ratio A” column in Table 1). Ratio A and Ratio B in Table 1 have an approximately normal distribution (*P* > 0.07). The mean of Ratio A is 5,236, with a standard deviation (SD) of 1,745, while the mean of Ratio B is only 2,414, with an SD of 1,148. Ratio B is calculated in the wider age range of 10–95 years, and the mean of Ratio B is significantly lower than the mean of Ratio A (*P* < 0.0001). These simple empirical figures illustrate that mortality rates decrease with age much faster during childhood than they increase during adulthood. It is obtained regardless of the model used or the shape of the total mortality trajectory.

In the present study, we first aimed to describe and explain decreases in mortality rate with age for congenital anomalies of the central nervous system (CACNS). We then aimed to explain decreases in total mortality rate with age during childhood. In addition, we investigated age-based changes in the trajectory of mortality for “diseases of the nervous system.”

### Congenital anomalies of central nervous system (CACNS)

**Figure 1:**
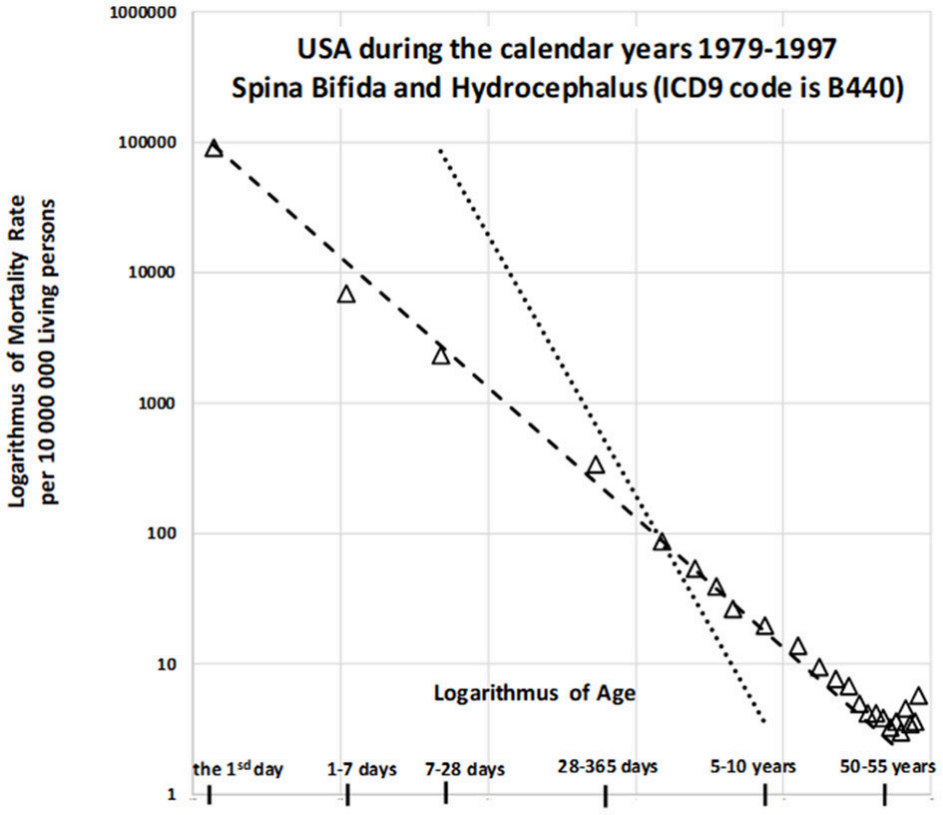
Decrease in mortality rate for spina bifida and hydrocephalus with age in US (1979–1997) on a log-log scale (24).

In the US, a deterministic relationship has been observed between mortality rate and age among those with CACNS. Previous studies have examined this trajectory in older patients between 1979 to 1997 (24), during which the 9th revision of the ICD was utilized. During this period, decreases in mortality with age were described for wide age intervals across two categories: “spina bifida and hydrocephalus” and “other deformities of the central nervous system.” The age trajectory for mortality due to “spina bifida and hydrocephalus” is shown in Figure 1.

The slope of the straight dashed line is −1 in Figure 1, in which the log–log scale is used, while the steeper dotted straight line has a slope of −2. The dashed straight line corresponds to the inverse relationship between mortality rate and age. The model is valid up to the relatively high age of 55 years, with the coefficient of determination *R*^2^ = 0.993. Similar results can be observed for the second category (“other deformities of central nervous system”), which exhibits a very high coefficient of determination [*R*^2^ = 0.999; (24)]. Such a deterministic relationship between the predictor and the response is not common in epidemiology, biology, or medicine. Two additional findings are important to the observations. First, congenital anomalies are dominant causes of death during the first year of life, especially in the first day and the first week, although the proportion of all deaths due to such anomalies decreases rapidly with age. [Specifically, for the nine countries indicated in Table 1, the proportion of deaths due to congenital anomalies and certain conditions originating during the perinatal period was 96% on the first day, 95% in the first week, and approximately 10% in the age interval of [5, 10) years]. Second, the inverse proportion may also describe the decrease in all-cause mortality with age (20, 21). If mortality due to CACNS decreases with age according to an inverse proportion, this may help to explain the overall decrease in mortality with age, although it remains to be determined whether this relationship is merely coincidental. If not, then the decrease in total mortality with age may be due to the extinction of more severe congenital impairments. Using data from the nine countries studied, we aimed to examine this hypothesis in order to provide crucial insights into high mortality rates during childhood.

### Large population

Zero deaths in a specific age group represents the principal obstacle to the construction of age trajectories of mortality from specific diseases. While zero deaths may occur due to CACNS within a specific age interval or calendar year over the age of 30 years, non-zero numbers may occur within the same interval in other calendar years. Including additional calendar years may help to overcome this obstacle, a strategy first utilized by the well-known astronomer and mathematician Edmond Halley (28, 29). In Ref. (30), Edmond Halley constructed a population life table for the city of Breslau using data collected between 1687 and 1691, based on his analysis of the numbers of births and deaths recorded in parish registers over several calendar years. This method enables the calculation of mortality rate in one age category based on the numbers of deaths and living persons for several years (24, 28, 29). It should be noted that standard epidemiologic and demographic interpretations of the specific value of the mortality rate within a specific age category are not self-evident. However, the interpretation is almost the same in all age categories, and the resulting age trajectory of mortality may be interpreted simply as a hallmark of aging. This method is also used in paleodemographic mortality analysis, in which a population and corresponding life table are constructed (28). Additionally, including more regions and calendar years within an analysis is expected to eliminate all factors other than age, allowing the impact of age to become more visible.

The WHO mortality database (26) registers the number of deaths in specific age categories in various countries. It is crucial to the construction of age trajectories of mortality that the database uses the following four age categories for the first year of life: [0, 24) h, [1, 7) days, [7, 28) days, and [28, 365) days. Unfortunately, these four categories are not used in all countries and in all calendar years. Cause of death is determined using the specific revision of the ICD in the database. Specific revisions of the ICD are used in different calendar years in different countries, with the 10th revision being the most recent. The calendar period used in each country corresponds to the period when the 10th revision is applied.

The present study used data collected in nine European countries and calendar periods (outer calendar years are shown in Table 1). The nine European countries represent approximately 97 million living persons in one calendar year (i.e., “large population”). Population size was comparable among countries, and there was no dominant region.

## Materials and methods

As the present study aimed to describe the dynamics of the relationship between age and mortality, as well as the trajectory of the decrease in mortality with age, we utilized the following definition of the force of mortality at exact age *x*:

(1) μ(x) = limh→0D(x+h)L(x) = −dS(x)dxS(x)≅DiLi.1(Bi−Ai),

where *D*(*x* + *h*) is the theoretical number of deaths in a small age interval [*x, x* + *h*), the theoretically infinitesimal increment *h* is positive, and age approaches exact age *x* “from the left.” *S*(*x*) represents the survival function (percentage of living people at age *x*), which is valid in principle: *S*(*x*) = 1 − *F*(*x*), where *F*(*x*) is the cumulative distribution function of the probability of death. The empirical value *Di* represents the number of deaths in a specific age interval [*Ai, Bi*), while *Li* represents the size of the population among which the deaths occurred. Changes in *Li* within an age interval [*Ai, Bi*) are empirically very small when compared with changes in *Di*. For this reason, the number of living people *Li* at age *Ai* can be used instead of the average number of living people. In other words, population *Li* goes through the “window” in time or through the age interval [*Ai, Bi*). The product *Li*·[*Bi*–*Ai*) in equation (1) represents the number of “person-years,” or the number of years lived by members of the population between ages *Ai* and *Bi*. The age trajectory of mortality is assumed to be an unknown theoretical curve, and it is constructed using the right side of equation (1). Mortality rate at an exact age, force of mortality, or simply mortality rate describes different age groups similarly to the way a decay constant describes the force of radioactive decay on different radionuclides. (In this comparison, the different radionuclides correspond to the various groups of patients).

To determine the detailed causes of death in specific age categories, we extracted the numbers of deaths in Denmark, Finland, Norway, Sweden, Austria, the Czech Republic, Hungary, Poland, and Slovakia from the “Morticd10_part1.txt” and “Morticd10_part2.txt” files of the WHO database (26). The four age intervals for the first year of life were used for the following calendar years, during which the ICD10 was also utilized: Denmark, 16 calendar years: 1994– Finland, 20 calendar years: 1996–2015; Norway, 20 calendar years: 1996–2015; Sweden, 19 calendar years: 1997–2015; Austria, 15 calendar years: 2002–2016; the Czech Republic, 22 calendar years: 1994–2015; Hungary, 20 calendar years: 1996–2015; Poland, 17 calendar years: 1999–2015; Slovakia, 14 calendar years: 1996–2014. The resulting dataset was regarded as the “large population,” which included all living individuals *Li* at the beginning of each age interval [*Ai, Bi*) and number of deaths *Di* in each age interval [*Ai, Bi*).

As the WHO database (26) is not user-friendly, we imported “txt” files into Microsoft (MS) Excel 2016. Moreover, a simple program was developed using Visual Basic for Applications to perform the following manipulations. MS Excel was used at the first level of processing, along with standard packages in R 3.3.2 for Windows. The level of statistical significance was set to 0.05 for all tests.

The database uses the following age intervals: [0, 24) h, [1, 7) days, [7, 28) days, [28, 365) days, [1, 2), [2, 3), [3, 4), [4, 5), [5, 10), [10, 15), [90, 95) years (26). The ICD10 three-digit code classification is used to the determination of the cause of death in the database. CACNS are described in Chapter XVII of the ICD10 [see also Table 3 in the Appendix; (27)]. A total of 5,984 deaths due to CACNS were found in the “large population” across all age intervals and all calendar years.

The database also contains the number of living people and the number of live births in the file labeled “Populations and live births.” Unfortunately, it uses a single age category within the wider age interval of [1, 5) years. Therefore, we obtained the numbers of living people from the U.S. Census Bureau database^1^, which uses 1-year age categories. The numbers of living people were summed for intervals after the age of 5 years.

Furthermore, the right side of equation (1) was used to calculate the mortality rate, providing the number of persons per year who died per 10,000,000 living people. The lowest number of deaths *Di* (*n* = 7) was observed for age interval of [85, 90) years, across all countries and all calendar years. [However, zero cases were observed in the last age interval [90, 95) years]. The resulting age trajectory of mortality due to CACNS was constructed for all calendar years using the ICD10 (26), four age categories in the first year of life, four age categories in the range of 1 to 5 years, and 5-year age categories in the range of 5–90 years. The arithmetic mean of the interval endpoints was used as a representative value for each age category. The time unit “1 year” was used in all age categories and in all calculations.

## Results

### Age trajectory of mortality due to CACNS

**Figure 2:**
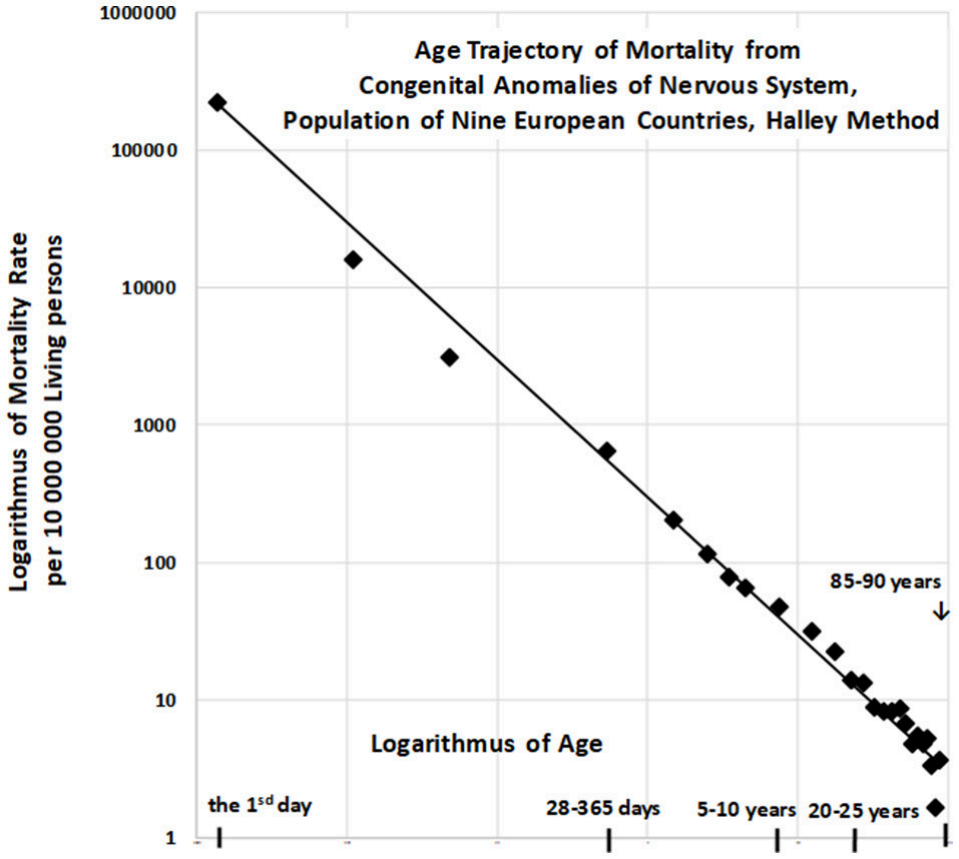
Age trajectory of mortality due to CACNS.

The resulting age trajectory of mortality due to CACNS is shown in Figure 2. The decrease in mortality rate is visually linear on the log–log scale. We first analyzed the decrease in mortality rate within the age interval of [0, 90) years. The linearity of the trajectory within the interval [0, 90) years was examined in the full quadratic model (2) using the method of least squares (LS):

(2) ln[μ(x)]=constant+γ.ln(x)+δ.ln(x).ln(x)

The null hypothesis for the quadratic element H_o_ : δ = 0 was not rejected (*P* > 0.75), while the parameter γ was significant (*P* < 0.0001). The *F*-test, in which the full model (2) does not provide a significantly better fit than the restricted model without the quadratic element, provided the same result. That is, the residual sum of squares was not significantly lower in the full model (*P* > 0.74). Consequently, we assumed that decreases in mortality with age were linear on the log–log scale, following which we developed a linear submodel (3):

(3) ln[μ(x)]=constant+γ.ln(x)

The two parameters ln[μ_1_] = constant, γ, the standard deviation of the two parameters, and the coefficient of determination R^2^ in the linear model (3) were calculated using the LS method for the age interval of [0, 90) years. The hypothesis that the residuals are age-independent was not rejected (*P* > 0.17), and our data further confirmed that the residuals are not U-shaped.

Linear regression analyses revealed that γ = −0.964, with a 95% CI of (−1.002, −0.926). The adjusted coefficient of determination ${\bar{R}}^{2}$ calculated for one predictor ln(*x*) and 25 age categories in the linear model (3) was 0.9911. Because the slope γ is close to the specific value −1, the null hypothesis H_o_: γ = −1 was examined using model (3), and was not rejected (*P* > 0.07). The specific value −1 for parameter γ corresponds to the inverse proportion between mortality rate and age. If γ = −1, then the following is formally valid:

(4) $$\begin{array}{l} \mu \left(x\right)=\mu_{1}/x\;or\;in\;the\;log-log\;scale\\ \;ln\left[\mu \left(x\right)\right]=ln\left(\mu_{1}\right)-ln\left(x\right) \end{array}$$

The parameter μ_1_ in model (4) can be estimated using equation (5) for *n* pairs of values ln[μ(*x*_*i*_)] and ln(*x*_*i*_), via the LS method:

(5) $$ln\left(\mu_{1}\right)=\sum \left\{ln\left[\mu \left(x_{i}\right)\right]+ln\left(x_{i}\right)\right\}\;/n$$

Furthermore, the standard coefficient of determination ${\text{R}}_{\text{b}}^{2}$ in model (4) can be described as follows:

(6) $$\begin{array}{l} R_{b}^{2}=1-SS_{resid}/SS_{Total}=1-\sum \{\ln\left[\mu \left(x_{i}\right)\right]-[\ln\left(\mu_{1}\right)\;\\ \;{-\;\ln\left(x_{i}\right)]\}}^{2}/\sum {\left\{\ln\left[\mu \left(x_{i}\right)\right]-\sum \ln\left[\mu \left(x_{i}\right)\right]/n\right\}}^{2} \end{array}$$

For the “large population”, the resulting value of ${\text{R}}_{\text{b}}^{2}$ was 0.9901 within the age interval of [0, 90) years. The inverse proportion (4) is a nested model that includes the two-parameter linear model (3). The null hypothesis that model (3) with two parameters does not provide a significantly better fit than model (4) with a single parameter was examined using a standard Fisher's test (*P* > 0.17). Consequently, the inverse proportion between mortality from CACNS and age was established for the age interval of [0, 90) years.

Similar results were observed for seven additional age intervals: [0, 5), [0, 10), [0, 15), [0, 85) years. Our model again revealed an inverse proportion between CACNS mortality and age. The results for each age interval are presented in Table 2. It should be noted that the results in a specific row in Table 2 correspond to events that are dependent on one another. The confidence intervals and *p*-values should thus be interpreted for single age intervals.

**Table 2:** Results of the linear regression calculated in the model (3) with two parameters and in the simple model (4).

**Upper age A**	**γ**	**Lower γ CI 95%**	**Upper γ CI 95%**	**R^2^**	**${\bar{R}}^{2}$**	**${\text{R}}_{\text{b}}^{2}$**	***F*-test**
5	−0.959	−1.048	−0.870	0.991	0.9900	0.9896	0.31
10	−0.952	−1.028	−0.876	0.9921	0.9909	0.9896	0.18
15	−0.943	−1.011	−0.876	0.992	0.9915	0.9889	0.09
20	−0.938	−0.998	−0.879	0.993	0.9922	0.9886	0.040
25	−0.942	−0.995	−0.888	0.994	0.9929	0.9898	0.040
30	−0.941	−0.989	−0.892	0.994	0.9936	0.9901	0.020
35	−0.946	−0.991	−0.900	0.994	0.9938	0.9910	0.020
40	−0.948	−0.990	−0.906	0.995	0.9942	0.9916	0.020
45	−0.948	−0.986	−0.909	0.995	0.9945	0.9919	0.010
50	−0.944	−0.981	−0.907	0.995	0.9946	0.9915	0.010
55	−0.944	−0.979	−0.910	0.995	0.9949	0.9918	0.002
60	−0.949	−0.983	−0.914	0.995	0.9948	0.9922	0.010
65	−0.949	−0.981	−0.916	0.995	0.9950	0.9924	0.001
70	−0.950	−0.981	−0.919	0.995	0.9952	0.9927	0.001
75	−0.949	−0.978	−0.919	0.996	0.9953	0.9926	0.003
80	−0.953	−0.982	−0.923	0.995	0.9950	0.9928	0.004
85	−0.964	−1.004	−0.924	0.991	0.9909	0.9899	0.07
90	−0.964	−1.002	−0.926	0.992	0.9911	0.9901	0.07

*Each row represents age interval [0, A), upper age **A** is in the first column. Slope γ, it's 95% confidence interval, coefficient of determination **R**^2^ and adjusted coefficient of determination ${\bar{R}}^{2}$ are calculated for one predictor and n points in the linear model (3), the coefficient of determination $R_{\text{b}}^{2}$ is calculated in the simple model (4) with single parameter: ln[μ(x)] = ln(μ_1_) – ln(x). p-value calculated in the standard **F-**test (the null hypothesis that model with two parameters (3) does not provide a significantly better fit than model (4) with single parameter) is in the last column. The quadratic element in the full quadratic model (2) is not significant in all age intervals (the null hypothesis: δ = 0 is not rejected with the p > 0.05)*.

### Subcategories of CACNS

The ICD10 includes eight detailed subcategories of CACNS, which provide key information regarding the biological background of the resulting age trajectory of CACNS mortality. Among all 5,984 deaths, the percentages of deaths due to each CACNS subtype were as follows: anencephaly and similar malformations (Q00), 12.2%; encephalocele (Q01), 4.9%; microcephaly (Q02), 7.7%; congenital hydrocephalus (Q03), 28.9%; other congenital malformations of the brain (Q04), 28.6%; spina bifida (Q05), 9.7%; other congenital malformations of the spinal cord (Q06),1.1%; other congenital malformations of the nervous system (Q07), 7.0%. More detailed information regarding CACNS can be found in the EUROCAT database (31).

**Figure 3:**
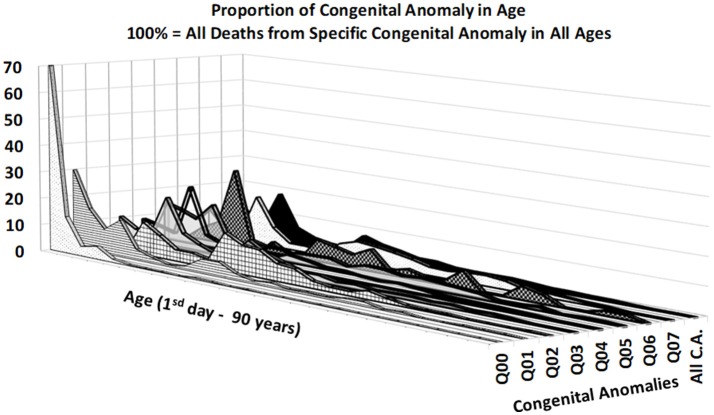
Percentage of deaths by CACNS subcategory across all ages.

**Figure 4:**
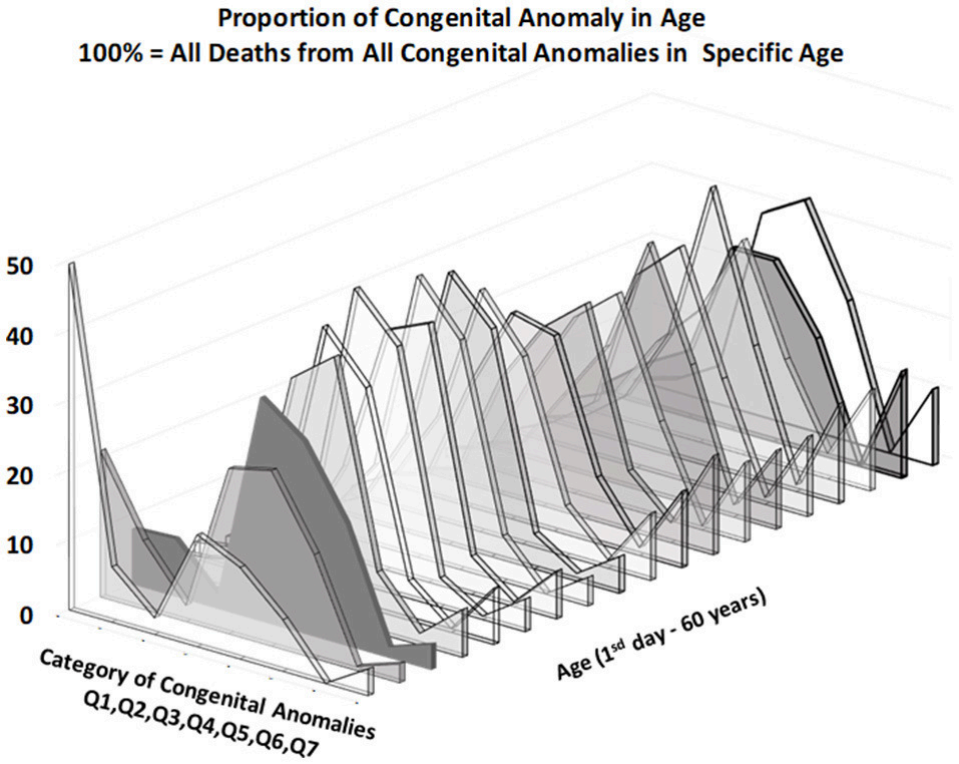
Percentage of deaths by CACNS subcategory in specific age categories.

Figures 3, 4 provide two different views of the impact of age on mortality in each of the eight subcategories.

At first glance, the category “anencephaly and similar malformations” (Q00:12.2% of all deaths due to CACNS) is exceptional. The category is dominant only in the first two age intervals: [0, 24) h and [1, 7) days (Figure 4). Specifically, 1,100 of all CACNS cases occur within the first day after birth, 544 of which fall within the Q00 category (49%), while 478 of all CACNS cases occur within the second age interval, 100 of which fall within the Q00 category (20.9%). In contrast, 7.9 and 3.2% of deaths due to Q00 occur during the third and fourth age intervals, respectively. In other words, the Q00 category, which usually includes more severe congenital impairments, gradually accounts for a lower proportion of CACNS deaths over the first year of life (Figure 3).

In contrast to category Q00, the proportions for categories Q01 through Q07 did not exhibit such dramatic extinction (Figure 4), as illustrated during the first week (i.e., the first two age intervals) and in intervals beyond the age of 50 years. Among all CACNS cases, 1,578 occurred within the first week after birth, at the following rates: anencephaly and similar malformations (Q00), 40.8%; encephalocele (Q01), 8.5%; microcephaly (Q02), 2.3%; congenital hydrocephalus (Q03), 18.3%; other congenital malformations of the brain (Q04), 16.7%; spina bifida (Q05), 9.6%; other congenital malformations of the spinal cord (Q06), 0.2%; other congenital malformations of the nervous system (Q07), 3.5%. A total 301 CACNS cases occurred after the age of 50 years, at the following rates: anencephaly and similar malformations (Q00), 0.3%; encephalocele (Q01), 3.7%; microcephaly (Q02), 5.3%; congenital hydrocephalus (Q03), 31.9%; other congenital malformations of the brain (Q04), 30.2%; spina bifida (Q05), 14.3%; other congenital malformations of the spinal cord (Q06), 2.3%; other congenital malformations of the nervous system (Q07), 12.0%. The extinction of the Q00 category supports the explanation that the exclusion of individuals with severe impairments caused decrease in mortality with age.

### Age trajectory of total mortality

**Figure 5:**
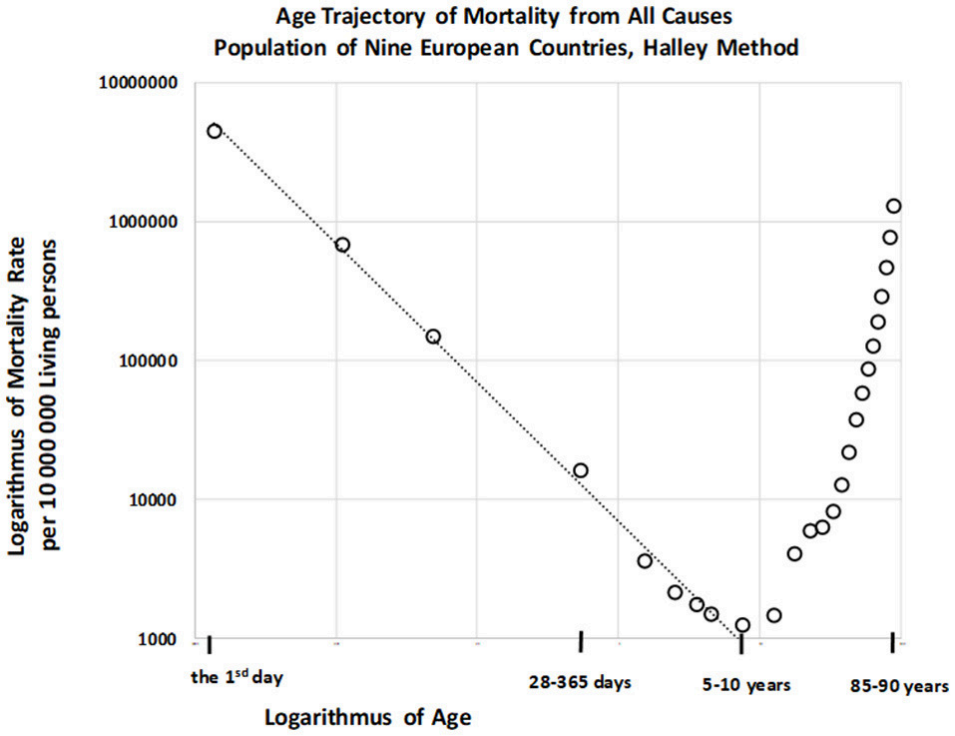
Age trajectory of total mortality.

Total numbers of deaths within each age category were also calculated for the “large population.” The resulting age trajectory of total mortality reached the minimal value within the age interval of [5, 10) years. Data were derived from 17,091,522 deaths across all age intervals, with 115,787 deaths occurring within the age interval of [0, 10) years. The resulting age trajectory of total mortality, which is approximately linear up to the age of 10 on the log–log scale, is presented in Figure 5.

The decrease in total mortality with age was analyzed within the age interval of [0, 10) years. Our results indicated that the null hypothesis for the quadratic element in model (2) was not rejected (*P* > 0.87), and that parameter γ was significant (*P* < 0.0001). The F-test that the full model (2) does not provide a significantly better fit than the restricted model without the quadratic element, in which the residual sum of squares was compared, revealed the same result. Decreases in all-cause mortality with age were linear on the log–log scale, and the linear model (3) was utilized for the following step. The two parameters ln[μ_1_], γ, the standard deviation of the two parameters, and the coefficient of determination R^2^ in the linear model (3) were calculated using the LS method. The hypothesis that the residuals are age-independent was not rejected (*P* > 0.92), and our analysis confirmed that the residuals are not U-shaped. Linear regression analyses using model 3 revealed that the slope γ was −0.997, with a 95% CI of (−1.054, −0.940). The adjusted coefficient of determination ${\bar{R}}^{2}$ for one predictor ln(*x*) and nine age categories in the linear model (3) was 0.9954.

Because the point estimation of the slope γ is close to the specific value −1 the null hypothesis H_o_: γ = −1 was tested using model (3), and was not rejected (*P* > 0.90). Furthermore, we examined whether model (4) provided a better fit for mortality rate than model (3). The resulting coefficient of determination ${\text{R}}_{\text{b}}^{2}$ as calculated using equation (6) was 0.9959, and the inverse proportion (4) represented a nested model in the two parametric linear models (3). A standard Fisher's test was used to examine the null hypothesis that model (3) does not provide a significantly better fit than model (4), which was not rejected (*P* > 0.90). These findings indicate that the inverse proportion between total mortality and age describes data within the age interval of [0, 10) years.

### Age trajectory of mortality from other causes

**Figure 6:**
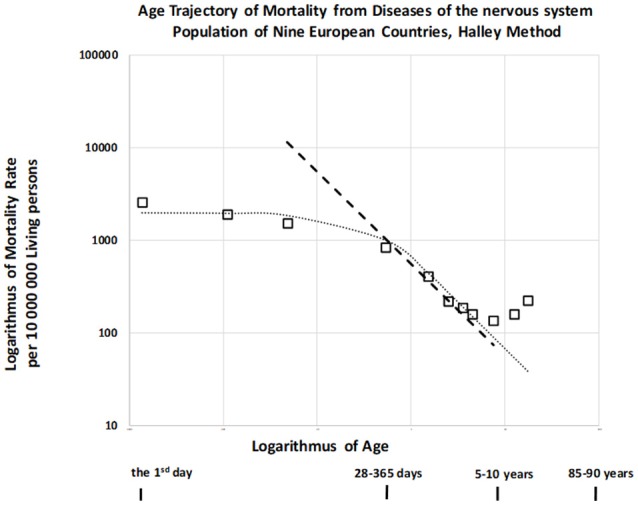
Age trajectory of mortality due to diseases of the nervous system (DNS).

It was previously indicated that mortality rate did not decrease with age or decreased slowly until the first or second year of life for other disease categories. Furthermore, this decrease occurred according to an inverse proportion at later ages (24) for infectious diseases, diseases of the nervous system (DNS), diseases of the respiratory system, and diseases of the digestive system (category names adopted from the ICD9). We also examined the age trajectory of mortality due to DNS, which are not usually associated with congenital impairment but may be biologically related to CACNS (codes G00-G99 in ICD10, see also Table 4 in the Appendix) (29). The resulting age trajectory of mortality due to DNS is presented in Figure 6.

Mortality was confirmed to be weakly dependent on age during the first year, and the slope of mortality decrease with age was approximately −1 in the age interval of [2, 10) years. (The dashed straight line has a slope of −1). This is a typical age trajectory for mortality, as described for other categories of disease unrelated to congenital impairment (24). Such a bent age trajectory of mortality can also be explained by the theory of congenital individual risk (TCIR) (23, 24).

At first, it is difficult to assume that any hypothetical individual development is switched on at the age of 1 or 2 years. Such hypothetical individual development does not affect mortality rates during the first 2 years in Figure 6, and it should cause a faster decrease in mortality at later ages. If the age trajectory of mortality corresponds to any individual development, then such a process is relatively weak during the first year, becoming significant after the age of 2 years. In contrast, the TCIR explains the mortality decrease with age based on the sequential extinction of more severe impairments. The TCIR can also be used to explain the bent age trajectory of mortality in Figure 6. These relationships are presented in the Appendix (in the section “Relationships describing bending trajectory of mortality from DNS”).

An important assumption of the TCIR is that *r* is age-independent, meaning that changes in congenital individual risk *r* are relatively small if they are evaluated with regard to the whole population. It does not mean that a possible development does not change the level of individual congenital anomalies. Furthermore, if age *x* is higher (*x* > 2), then the exponential term in the squared brackets can be neglected in equation (7), and the inverse proportion is valid. Consequently, this theoretical relationship (7) can also explain the bending of the age trajectory of mortality in Figure 6, where it corresponds to the dotted curve.

Why may congenital risks be limited in these categories of diseases? Why may the limit be higher for congenital anomalies? TCIR explains these as the result of the administrative selection of severe cases to the categories related to congenital anomalies. For example, if an individual case of death is related to the nervous system and no visible congenital impairment is diagnosed, then such hypothetical congenital impairment is latent. (The category of death does not automatically contain cases with severe and visible congenital impairments). Consequently, the spectrum of *r* should be limited by some lower maximum in the set of diseases unrelated to congenital anomalies. This explanation may account for the empirical age trajectories of mortality in Figure 6. In this case, *r*_max_ = 2.90 per year and μ_1_ = 686 per year per 10,000,000 living people. Model (7) is used here to calculate the two values used in the construction of the dotted curve in Figure 6. These two values were determined numerically by minimizing the residual sum of squares with the resulting coefficient of determination, which was equal to 0.966.

In principle, no such limit exists for congenital individual risk *r* in the all-cause category, which may in reality be very high. The limit *r*_max_ corresponds to specific category definitions in the ICD, and may correspond to specific usage of the ICD in different countries. This may explain the decrease in total mortality rate with age according to an inverse proportion following the first day of life (Figure 3), which may simultaneously be age-independent during the first two years of life. It should be emphasized that the inverse proportion is also valid for total mortality and CACNS from the first day of life, and that the coefficients of determination are very high. The coefficient of determination is 0.996 for total mortality in the age interval of [0, 10) years and 0.990 for CACNS in the age interval of [0, 90) years.

## Discussion

The first explanation which comes to mind may be that mortality rate decreases with age due to the biological development of each individual. However, we presented entirely different explanation. We hypothesized that this decrease can be explained by the extinction of more sever patients. According to TCIR, the shape age trajectory of mortality may be a hallmark of the distribution of congenital impairments at the moment of birth.

The age trajectory of mortality due to CACNS was constructed using the “large population” and does not describe a standard biologically born cohort. In addition, it does not provide a cross-sectional description in a specific calendar year. Large populations and corresponding age trajectories of mortality represent a compromise between a standard cross-sectional study (transversal study) in a single calendar year and a longitudinal study of a cohort born in a single year. The periods when the same revision of ICD was used were relatively short (Table 1). Thus, the difference between longitudinal and cross-sectional studies can be partially neglected in the “large population” for the shorter age interval of [0, 15) years. The length of the age interval [0, 15) years corresponds to the length of the calendar period in which the “large population” was constructed. It can be also expected that variations in the level of health care or external influences are smaller during this 15-year interval. The age interval of [0, 15) years has other advantages because it corresponds to the age intervals in which total mortality decreases with age (Table 1). Furthermore, the age interval of [0, 15) years also eliminates the following problem associated with the TCIR. The TCIR assumes that changes in the denominator *S*(*x*) with age *x* in equation (1) can be neglected against changes in the numerator –*dS*(*x*)/*dx* with age *x*. The assumption is empirically valid only up to the age of 25 years (*S*(*x*) = 0.989 for 25 years in the “large population”). If all objections are taken into account, then the age interval [0, 15) years (Table 2, third row) may be more appropriate than the full interval of [0, 90) years.

A total of 4,632 deaths from CACNS are registered in the age interval [0, 15) years. The hypothesis that the residuals are age-independent was not rejected (*P* > 0.18), and the residuals were not U-shaped. Linear regression analyses using model (3) revealed that γ = −0.943, with a 95% CI of (−1.011, −0.876). The adjusted coefficient of determination ${\bar{R}}^{2}$ for one predictor ln(*x*) and 10 age categories was 0.992. Furthermore, the coefficient of determination ${\text{R}}_{\text{b}}^{2}$ calculated based on model (4) was 0.989, and the null hypothesis that model (3) does not provide a significantly better fit than model (4) with a single parameter was not rejected (*P* > 0.09). Consequently, the inverse proportion between mortality from CACNS and age was also modeled successfully within the age interval of [0, 15) years.

All empirical observations in the study were made using the Halley method, in which age is regarded as the dominant factor, while all other factors are regarded as smaller, with different directions of influence in different regions. In this method, factors other than age are expected to eliminate on another as more regions and calendar years are included. The resulting age trajectory of mortality due to CACNS supports the assumption in Figure 2. The mortality rate due to CACNS may decrease with age according to an inverse proportion up to the age of 90 years, and it is difficult to accept the notion that such decreases represent individual developmental processes. The spectrum of CACNS in the born population is highly variable. While some individuals are born with severe impairments that lead to death during the first hours or week of life, others with less severe impairments can survive to old age. According to the TCIR, the resulting mortality decrease with age can be explained by the sequential extinction of individuals with severe impairments. This assumption is supported by the extinction of category Q00, as demonstrated in Figures 3, 4.

The TCIR assumes that the level of severity is approximately age-independent. However, this explanation does not entirely reject the possibility that some positive or negative changes in congenital anomalies occur with age. The severity of individual impairment was compared to all other levels of impairment in the whole population. The inverse proportion between mortality rate and age is explained by the following assumption: “The more severe the malformation, the less frequently it occurs in the born population” (or by the relationship *f* (*r*) ≅ constant/*r* where *r* represents the congenital risk of death). Such a speculative distribution may be the result of selection during pregnancy or previous generations.

Most importantly, our empirical evidence indicates that the total mortality rate also decreases with age according to an inverse proportion, with a high coefficient of determination (0.996). The coincidence between the age trajectories of all-cause and CACNS-related mortality may indicate that the overall decrease in mortality after birth is also due to the extinction of individuals with more severe impairments.

Our findings indicated that decreases in mortality rates for other diseases with age occur more slowly or are age-independent during the first year, following which the rates decrease according to an inverse proportion. Such bent age trajectories of mortality can also be explained using the TCIR. If a relatively small upper limit of congenital individual risks exists in a specific group of diseases, then the theoretical two-parameter relationship (7) may fit the empirical data. This is illustrated in the DNS category in Figure 6, strongly supporting the TCIR.

The TCIR is characterized by two basic assumptions: (a) *r* is age-independent, and (b) its frequency function *f* (*r*) in the born population is approximately *f* (*r*) ≅ constant/*r*. It is empirically difficult to verify the second assumption because the actual values of *r* are unknown, and it is difficult to determine individual values of *r*. However, if the first assumption that *r* is age-independent is valid, then the mortality rate is formally the Laplace transform of the product *r.f(r*). Consequently, the empirical inverse proportion between mortality rate and age may be a hallmark of the relationship described by *f* (*r*) ≅ constant/*r*. The total mortality rate also decreases with age according to an inverse proportion, strongly suggesting that decreases in total mortality are caused by the extinction of individuals with more severe impairments. Further studies are required to determine whether these relationships are observed in other countries.

### Consequences in clinical practice

Mortality rate from the majority of diseases sharply decreases with age and malignant neoplasms are important exception from the observation. Mortality rate from malignant neoplasms is age-independent from the first month up to the age of 25 years (24). According to TCIR the majority of deaths up to the age of 10 years is related to congenital impairment and the decrease of mortality rate with age is a demonstration of population heterogeneity. The explanation of the mortality decrease with age leads to the existence of latent congenital defects. Such latent congenital defects cause death even no congenital impairment is observed. For a wonder cases put in the first chapter of ICD 10 “Certain infectious and parasitic diseases” could be associated with a congenital defect. Namely, if influenza is determined as the cause of death then latent congenital impairment could be actual cause of death. Other epidemiological studies also support that significantly higher proportion of individuals with an inherited predisposition are among dead [e.g., (32)]. These findings show that there are two basic children's patients in clinical practice. The bigger group of patients is without congenital impairment and the smaller group is characterized by congenital defects. Such congenital defects could be latent despite they are primary causes of death. According to TCIR the second group is relatively small if compared with the whole population but it is dominant among dead up to the age of 10 years.

All results were derived from the WHO mortality database (26). The division of the first year of life into four age categories was the key for our observations, as this detailed division was necessary for the assumptions of our analyses. Age of death is very reliable, and exact information is available for individual cases in developed countries. If more detailed categories of age are used, then the resulting age trajectory of mortality will inevitably be more detailed. In principle, detailed information regarding the age of death should be included in health information systems in developed countries. This information is wasted in the construction of databases if individual cases are contained within wider age categories.

## Data availability

All results are based on published data, and the data are presented here as a supplement.

## Author contributions

JD designed the study. PM and HH contributed to the literature searches. JD contributed in preparing the draft of the manuscript. All authors read and approved the final manuscript.

### Conflict of interest statement

The authors declare that the research was conducted in the absence of any commercial or financial relationships that could be construed as a potential conflict of interest.

## Footnotes

^1^The U.S. Census Bureau, USCB (2017). Available online at: https://www.census.gov/data-tools/demo/idb/informationGateway.php (Accessed May 22, 2017).
