# Supplementary material for: Modeling the Age-Associated Decrease in Mortality Rate for Congenital Anomalies of the Central Nervous System Using WHO Metadata From Nine European Countries
Source: Front Neurol. 2018 Jul 19;9:585. doi: 10.3389/fneur.2018.00585 (PMC6067090; doi:10.3389/fneur.2018.00585)
Supplement: Supplementary file 1 [file Data_Sheet_1.pdf]

Relationships describing bending trajectory of mortality from DNS

If some maximal limit bounds congenital individual risks in the born population, then the following approximation may be valid according to the TCIR (Dolejs, 2001; Dolejs, 2003):

$$\mu(x) = \int_0^{r_{max}} c.e^{(-r.x)}dr = c.\left[\frac{e^{(-r.x)}}{-x}\right]_0^{r_{max}} = c.\frac{e^{(-r_{max}.x)}}{-x} - c.\frac{1}{-x} = \frac{\mu_1}{x}.\left[1 - e^{(-r_{max}.x)}\right] \qquad (7)$$

In the present study, *Di* decreased by three orders of magnitude, and *S*(*x*) was higher than 0.98 at the age of 10 years. Consequently, the changes in the denominator (number of living persons) with age can be neglected due to the changes in the numerator of equation (1). Based on the TCIR, we simultaneously assumed that the distribution of individual congenital risk *r* can be approximated by the relationship *f*(*r*) = *c*/*r*. Because the numerator is the partial derivative of *S*(*x*) with respect to the variable *x*, the variable *r* is only in the exponential term. (Variable *r* represents the denominator in the frequency function *f*(*r*), and it is also the result of the partial derivative in the numerator.) Furthermore, if the product $r_{max}.x$ is small in equation (7), then the theoretical mortality rate is approximately constant according to the following relationship (8) (Dolejs, 2001; Dolejs, 2003):

$$\mu(x) = \frac{\mu_1}{x}.\left[1 - e^{(-r_{max}.x)}\right] \cong \frac{\mu_1}{x}\left[1 - (1 - r_{max}.x)\right] = \mu_1.r_{max} \qquad (8)$$

If a time unit of 1 year is used, and if r is represented per year, then the product $r_{max}.x$ is unitless. The approximation (8) is valid if the product $r_{max}.x$ is very small, relative to 1.

Table 3
Congenital malformations of the nervous system according ICD10, Chapter XVII, codes: Q00-Q07

Q00 Anencephaly and similar malformations
Q00.0 Anencephaly, Acephaly, Acrania, Amyelencephaly, Hemianencephaly, Hemicephaly,
Q00.1 Craniorachischisis
Q00.2 Iniencephaly
Q01 Encephalocele,
Incl.: encephalomyelocele, hydroencephalocele, hydromeningocele, cranial, meningocele, cerebral, meningoencephalocele,
Excl.: Meckel-Gruber syndrome (Q61.9)
Q01.0 Frontal encephalocele
Q01.1 Nasofrontal encephalocele
Q01.2 Occipital encephalocele
Q01.8 Encephalocele of other sites
Q01.9 Encephalocele, unspecified
Q02 Microcephaly
Incl.: Hydromicrocephaly Micrencephalon Excl.: Meckel-Gruber syndrome (Q61.9)
Q03 Congenital hydrocephalus
Incl.:hydrocephalus in newborn Excl.: Arnold-Chiari syndrome (Q07.0), hydrocephalus: acquired NOS (G91.-), acquired, of newborn (P91.7) due to congenital toxoplasmosis (P37.1), with spina bifida (Q05.0-Q05.4)
Q03.0 Malformations of aqueduct of Sylvius, Aqueduct of Sylvius: anomaly, obstruction, congenital stenosis
Q03.1 Atresia of foramina of Magendie and Luschka, Dandy-Walker syndrome
Q03.8 Other congenital hydrocephalus
Q03.9 Congenital hydrocephalus, unspecified
Q04 Other congenital malformations of brain
Excl.: cyclopia (Q87.0), macrocephaly (Q75.3)
Q04.0 Congenital malformations of corpus callosum
Agenesis of corpus callosum
Q04.1 Arhinencephaly
Q04.2 Holoprosencephaly
Q04.3 Other reduction deformities of brain, Absence, Agenesis, Aplasia, Hypoplasia, Agyria, Hydranencephaly, Lissencephaly, Microgyria, Pachygyria, Excl.: congenital malformations of corpus callosum (Q04.0)
Q04.4 Septo-optic dysplasia
Q04.5 Megalencephaly
Q04.6 Congenital cerebral cysts, Porencephaly, Schizencephaly. Excl.: acquired porencephalic cyst (G93.0)
Q04.8 Other specified congenital malformations of brain, Macrogyria
Q04.9 Congenital malformation of brain, unspecified, Congenital: anomaly deformity disease or lesion, multiple anomalies
Q05 Spina bifida
Incl.: hydromeningocele (spinal), meningocele (spinal), meningomyelocele, myelocele, myelomeningocele, rachischisis, spina bifida (aperta)(cystica) , syringomyelocele, Excl.: Arnold-Chiari syndrome (Q07.0), spina bifida occulta (Q76.0)
Q05.0 Cervical spina bifida with hydrocephalus
Q05.1 Thoracic spina bifida with hydrocephalus, Spina bifida: dorsal, thoracolumbar
Q05.2 Lumbar spina bifida with hydrocephalus, Lumbosacral spina bifida with hydrocephalus
Q05.3 Sacral spina bifida with hydrocephalus
Q05.4 Unspecified spina bifida with hydrocephalus
Q05.5 Cervical spina bifida without hydrocephalus
Q05.6 Thoracic spina bifida without hydrocephalus, Spina bifida: dorsal NOS, thoracolumbar NOS
Q05.7 Lumbar spina bifida without hydrocephalus, Lumbosacral spina bifida NOS
Q05.8 Sacral spina bifida without hydrocephalus
Q05.9 Spina bifida, unspecified
Q06 Other congenital malformations of spinal cord
Q06.0 Amyelia
Q06.1 Hypoplasia and dysplasia of spinal cord, Atelomyelia, Myelatelia, Myelodysplasia of spinal cord
Q06.2 Diastematomyelia
Q06.3 Other congenital cauda equina malformations
Q06.4 Hydromyelia , Hydrorachis
Q06.8 Other specified congenital malformations of spinal cord
Q06.9 Congenital malformation of spinal cord, unspecified, Congenital: anomaly, deformity, disease or lesion
Q07 Other congenital malformations of nervous system,
Excl.:, familial dysautonomia [Riley-Day] (G90.1), neurofibromatosis (nonmalignant) (Q85.0)
Q07.0 Arnold-Chiari syndrome
Q07.8 Other specified congenital malformations of nervous system, Agenesis of nerve, Displacement of brachial plexus, Jaw-winking syndrome, Marcus Gunn syndrome
Q07.9 Congenital malformation of nervous system, unspecified

Table 4
Diseases of the nervous system according ICD10, Chapter VI, codes: G00-G99

Diseases of the nervous system	G00-G99
Inflammatory diseases of the central nervous system	G00-G09
Systemic atrophies primarily affecting the central nervous system	G10-G13
Extrapyramidal and movement disorders	G20-G26
Other degenerative diseases of the nervous system	G30-G32
Demyelinating diseases of the central nervous system	G35-G37
Episodic and paroxysmal disorders	G40-G47
Nerve, nerve root and plexus disorders	G50-G59
Polyneuropathies and other disorders of the peripheral nervous system	G60-G64
Diseases of myoneural junction and muscle	G70-G73
Cerebral palsy and other paralytic syndromes	G80-G83
Other disorders of the nervous system	G90-G99
